# Sexual Function and Associated Factors in Postmenopausal Women

**DOI:** 10.1055/s-0041-1735128

**Published:** 2021-08-30

**Authors:** Socorro Rejany Sales Silva Trento, Alberto Madeiro, Andréa Cronemberger Rufino

**Affiliations:** 1 Programa de Pós-Graduação em Saúde e Comunidade, Universidade Federal do Piauí, Teresina, PI, Brazil

**Keywords:** sexuality, sexual dysfunctions, psychological, climateric, women's health, postmenopause, sexualidade, disfunções sexuais psicogênicas, climatério, saúde da mulher, pós-menopausa

## Abstract

**Objective** To assess the sexual function and associated factors in postmenopausal women.

**Methods** This a descriptive, cross-sectional study with 380 women aged 40 to 65 years, users of public health services in 2019. Questionnaires were applied on demographic characteristics, on climacteric symptoms (menopause rating scale) and on sexual function (sexual quotient, female version). Bivariate and multiple analyses by logistic regression were performed, with adjusted odds ratios (OR _ad_ ) and 95% confidence intervals (95%CIs).

**Results** More than half (243/64%) of the participating women were at risk of sexual dysfunction, with lower scores in the domains of sexual desire and interest, comfort, orgasm, and satisfaction. Women with a partner (OR _ad_ 2.07; 95%CI 1.03–4.17) and those who reported sleep problems (OR _ad_ 2.72; 95%CI 1.77–4.19), depressed mood (OR _ad_ 2.03; 95%CI 1.32–3.10), sexual complaints (OR _ad_ 8.16; 95%CI 5.06–13.15), and vaginal dryness (OR _ad_ 3.44; 95%CI 2.22–5.32) showed greater chance of sexual dysfunction.

**Conclusion** There was a high prevalence of sexual dysfunction, with the influence of conjugality and climacteric symptoms on sexual function.

## Resumo

**Objetivo** Avaliar a função sexual e fatores associados em mulheres na pós-menopausa.

**Métodos** Este é um estudo descritivo, transversal, com 380 mulheres de 40 a 65 anos, usuárias de serviços de saúde públicos em 2019. Foram aplicados questionários sobre características demográficas, sobre sintomas climatéricos ( *menopause rating scale* ) e sobre a função sexual (Quociente Sexual, versão feminina). Análises bivariada e múltipla por regressão logística foram realizadas, com cálculo de *odds ratio* ajustado (OR _aj_ ) e intervalos de confiança de 95% (ICs95%).

**Resultados** Mais da metade (243/64%) das mulheres participantes no estudo apresentou risco de disfunção sexual, com escores mais baixos dos domínios desejo e interesse sexual, conforto, orgasmo e satisfação. Mulheres com companheiro (OR _aj_ 2,07; IC95% 1,03–4,17) e aquelas que relataram problemas de sono (OR _aj_ 2,72; IC95% 1,77–4,19), ânimo depressivo (OR _aj_ 2,03; IC95% 1,32–3,10), queixas sexuais (OR _aj_ 8,16; IC95% 5,06–13,15) e ressecamento vaginal (OR _aj_ 3,44; IC95% 2,22–5,32) apresentaram maior chance de disfunção sexual.

**Conclusão** Houve prevalência elevada de disfunção sexual, com influência da conjugalidade e dos sintomas climatéricos sobre a função sexual.

## Introduction

Sexual function results from the influence of organic, psychological, and interpersonal factors. Psychophysiological or interrelational problems interfere with the sexual function and offer obstacles to sexual satisfaction, which result in sexual dysfunctions. 1 The sexual function reflects the phases of sexual response, and its assessment can be used as a measure of sexual well-being. 2 Sexual health care includes the assessment of sexual function, which must also be incorporated into actions developed in basic health care. 2 3

There is a consensus that the human sexual response presents a predictable sequence of somatic and psychological changes, although it may differ between men and women. 4 The biological aspect of the female sexual response was initially described in four linear and successive phases, 5 being subsequently characterized by a three-phase model composed of desire, excitement, and orgasm. 6 In 2001, the value of interpersonal relationships in women's sexuality was highlighted, with the proposition of a female sexual function model with a circular trajectory in which emotional stimuli and relationships play a fundamental role. 7

Multiple causes can impair female sexual response, leading to sexual dysfunction. Data from a study that evaluated the sex life of Brazilians pointed out, however, that women in a higher age group were the most dissatisfied with their sex life. 8 The prevalence of sexual dysfunction in women in menopause is high, ranging from 35.9 to 67% among women from different Brazilian locations. 9 10 11 12 Urogenital changes resulting from hypoestrogenism have the potential to reverberate in postmenopausal sexual function, 13 changing the domains of desire, excitement, and orgasm, and impacting interpersonal functioning and quality of life. 14 15 16

Estrogen interferes with the female sexual function by having a central action in the hypothalamus and preoptic area that leads to a positive influence on motivation, mood, and sexual desire. It also acts directly on the vulva and vagina, increasing genital blood flow and vaginal lubrication. In this way, estrogen works by improving sexual desire and arousal. 17 Thus, hypoestrogenism can result in less sexual responsiveness by acting on the central nervous system and peripheral tissues. 17 18 However, observational studies have shown that the relationship with the partner and the state of physical and mental health were more strongly associated with sexual function than the serum estrogen levels. 18

Some authors point out that poor vaginal lubrication can result in dyspareunia and, consequently, have an impact on the decrease of sexual desire. This can discourage women from participating in sexual relations with their partners or it can be an interference factor in the other phases of female sexual response. 19 20 The correlation observed between typical symptoms of hypoestrogenism and sexual dysfunction in postmenopausal women reinforces this hypothesis, especially when somato-vegetative and urogenital symptoms are reported. 20 In addition, the intensity of somato-vegetative and urogenital symptoms has strong association with worse sexual function. 12

Considering the increase in life expectancy as a current trend, most women spend about a third of their existence in the postmenopausal period. 13 Thus, sexuality must also receive full attention in the climacteric, since it represents one of the pillars of quality of life and a marker for women's general health. In view of the disorders that sexual dysfunctions may cause, the aim of the present study was to investigate the sexual function and associated factors of postmenopausal women.

## Methods

This is a descriptive and cross-sectional study with postmenopausal women between 40 and 65 years old, residing in the urban region of Teresina, Piauí, and attended at Basic Health Units (BHUs). Piauí is a Brazilian state, located in the Northeast region. For the diagnosis of menopause, the clinical criterion of amenorrhea for a minimum period of 12 months was used. Women who reported absence of sexual practice in the last 6 months of the survey, and those with cognitive and/or neurological changes that could make it difficult to complete the questionnaire were excluded from the study.

For the sample calculation, simple random sampling without replacement was used. A universe of 30,164 women was used, according to the number of women registered in Primary Care in Teresina, within the age group established in the study. A 95% confidence interval was adopted in the estimates and variance based on the prevalence of sexual dysfunction of 49.6%, 11 with a 5% error in the parameters to be estimated. The final sample size was 380 women.

The BHU that participated in this study were selected using simple random sampling, limited to 4 BHU in each area of the city (Center/North, South, East/Southeast) until completing the minimum sample size required for the study. Data collection took place from September 2018 to September 2019. The eligible women were randomly selected and invited to participate in the research, and the informed consent form (ICF) was filled out in a reserved room.

Three instruments were used: 1. questionnaire with sociodemographic data; 2. questionnaire on menopause symptoms; and 3. the sexual quotient, female version (SQ-F). First, the following sociodemographic data were collected: age range (40–49; 50–59; 60–65), skin color (black; brown; indigenous; white; yellow), education (years: no; up to 11; >11), professional occupation (with occupation; with no occupation), family income (wages: no; up to 2; > 2), conjugality (no; yes), and use of hormone therapy (no; yes).

Second, a questionnaire on menopause symptoms was applied, known as the menopause rating scale (MRS), already validated and translated in Brazil. 21 It presents 11 questions in domains of somato-vegetative symptoms (hot flashes, cardiac malaise, sleeping, muscle and joint problems), urogenital (bladder, sexual problems and vaginal dryness) and psychological (depressed mood, irritability, anxiety, physical and mental exhaustion), with answers to each question classified on a severity scale, ranging from zero (absence of symptom) to four (very severe symptom). For the total score, the scores of all domains are added, classifying women as asymptomatic (< 4 points), with mild symptoms (from 5–8 points), with moderate symptoms (9–15 points) and with severe symptoms (> 16 points).

Finally, to investigate the sexual function, the SQ-F, developed for the Brazilian population, was used. 22 The questionnaire consists of 10 self-response questions with domains on sexual desire and interest, foreplays, personal arousal, and attunement with the partner, comfort, orgasm, and satisfaction. Each question is answered on a scale of 0 to 5, with a score obtained by adding the points of all questions (multiplied by 2), and a final score ranging from 0 to 100. A total score equal to or below 60 is considered a risk of dysfunction sexual. 22

The data were organized in Windows Office Excel 2010 spreadsheets (Microsoft Corp., Redmond, WA, USA) and later analyzed using the IBM Statistical Package for the Social Sciences (SPSS) for Windows, Version 20.0 (IBM Corp., Armonk, NY USA), and R-Project version 3.0.2 applications (R Foundation for Statistical Computing, Vienna, Austria). The MRS data were analyzed using dimensions that involve menopausal symptoms. The SQ-F information was evaluated by adding the individual scores of the women to assess sexual performance and track sexual dysfunctions. A bivariate analysis was performed using the Pearson chi-square test or the Student *t* test to associate sociodemographic variables and climacteric symptoms with women's sexual function. To check the abnormality of the quantitative variables, the Kolmogorov-Smirnov test was applied. The effect of the independent variables on the response variable (SQ-F score ≤ 60) was assessed by multiple logistic regression, with adjusted odds ratios (ORs _ad_ ) and 95% confidence intervals (95%CIs). For the inclusion of variables in the logistic model, the criterion of *p* < 0.20 was established in the bivariate analysis. In all tests, *p* < 0.05 was adopted as a significant difference or association.

The study was approved by the Research Ethics Committee of the Federal University of Piauí (CAAE 95272918.0.0000.5214), in compliance with Resolution 466/12 of the National Health Council.

## Results

Most women were in the 50 to 59 years (68.4%) age group, had black/brown skin color (85.3%), up to 11 years of schooling (89.2%), family income up to 2 minimum wages (77.4%) and lived with a partner (90.3%). Only 5.8% of them used hormone therapy ( Table 1 ).

**Table 1:** Characterization of women according to sociodemographic variables

Characteristics	n	%
Age range (years)		
40–49	48	12.6
50–59	260	68.4
60–65	72	18.9
Skin color		
Yellow/indigenous	8	2.1
White	48	12.6
Black/brown	324	85.3
Education (years)		
No	18	4.7
Up to 11	339	89.2
> 11	23	6.1
Professional occupation		
With occupation	192	50.5
With no occupation	188	49.5
Family income (wages)		
No	14	3.7
Up to 2	294	77.4
> 2	71	18.7
Do not know how to inform	1	0.3
Conjugality		
No	37	9.7
Yes	343	90.3
Use of hormone therapy		
No	358	94.2
Yes	22	5.8

More than half (64%) of the women had a total SQ-F score lower than 60 points, characterizing sexual performance and satisfaction ranging from null to regular, and, therefore, being screened for the presence of sexual dysfunction. The mean total score of the SQ-F was 52.12 points, and the most affected domains were sexual desire and interest (43.1%), followed by comfort (43.4%) and orgasm and satisfaction (46.6%) ( Table 2 ).

**Table 2:** Sexual function of women, according to the sexual quotient, female version (SQ-F)

**Sexual performance and satisfaction**	**Points**	**n**	**%**
Good to excellent	82–100	45	11.8
Regular to good	62–80	92	24.2
Unfavorable to regular	42–60	110	29.0
Bad to unfavorable	22–40	105	27.6
Null to bad	0–20	28	7.4
Total	–	380	100.0
**Domains**	**Scores**	**Average**	**Level (%)**
Sexual desire and interest	0–15	6.47	43.1
Foreplay	0–5	2.92	58.3
Personal excitement/alignment with partnership	0–10	5.27	52.7
Comfort	0–10	4.34	43.4
Orgasm and satisfaction	0–10	4.66	46.6
Total score	0–100	52.12	–

As for the symptoms in the MRS, 98.4% of women presented some symptoms, and in 38.2% of them the symptoms were classified as severe (data not shown in the table). The most reported symptoms were muscle/joint problems (75.3%), anxiety (67.2%), and sexual problems (64.7%), with a predominance of the urogenital domain (78.9%) ( Table 3 ).

**Table 3:** Association of the risk of sexual dysfunction with the domains and symptoms of the menopause rating scale

Variables	Present symptoms	Risk of sexual dysfunction (SQ-F ≤ 60)	Absence of sexual dysfunction (SQ-F ≥ 62)	*p* -value *
	n (%)	n (%)	n (%)	
Domains				
Psychological	271 (71.3)	191 (70.5)	80 (29.5)	< 0.001
Somatic	295 (77.6)	209 (70.8)	86 (29.2)	< 0.001
Urogenital	300 (78.9)	218 (72.7)	82 (27.3)	< 0.001
Symptoms				
Hot flashes	221 (58.2)	157 (71.0)	64 (29.0)	< 0.001
Cardiac malaise	175 (46.0)	125 (71.4)	50 (28.6)	< 0.001
Sleeping problems	212 (55.8)	157 (74.1)	55 (25.9)	< 0.001
Depressed mood	195 (51.3)	140 (71.8)	55 (28.2)	< 0.001
Irritability	222 (58.4)	159 (71.6)	63 (28.4)	< 0.001
Anxiety	255 (67.1)	177 (69.4)	78 (30.6)	< 0.001
Physical and mental exhaustion	212 (55.8)	157 (74.1)	55 (25.9)	< 0.001
Sexual problems	246 (64.7)	198 (80.5)	48 (19.5)	< 0.001
Bladder problems	69 (18.2)	50 (72.5)	19 (27.5)	< 0.001
Vaginal dryness	214 (56.3)	163 (76.2)	51 (23.8)	< 0.001
Muscle/joint problems	286 (75.3)	186 (65.0)	100 (35.0)	< 0.001

* Pearson's chi-square test.

Table 4 shows that the presence of sexual dysfunction was associated with an absence of family income ( *p* = 0.041) and the presence of conjugality ( *p* = 0.048). There was also a statistically significant association between the psychological, somato-vegetative and urogenital domains with the presence of sexual dysfunction ( *p* < 0.001). All symptoms on the MRS scale were associated with the risk of sexual dysfunction ( *p* < 0.001) ( Table 3 ).

**Table 4:** Association between the risk of sexual dysfunction and sociodemographic variables

Characteristics	Risk of sexual dysfunction (SQ-F ≤ 60)	Absence of sexual dysfunction (SQ-F ≥ 62)	*p* -value *
	n (%)	n (%)	
Age range (years)			
40–49	28 (58.3)	20 (41.7)	0.620
50–59	170 (65.4)	90 (34.6)	
60–65	45 (62.5)	27 (37.5)	
Skin color			
Yellow/indigenous	6 (75.0)	2 (25.0)	0.725
White	32 (66.7)	16 (33.3)	
Black/brown	205 (63.3)	119 (36.7)	
Education (years)			
No	11 (61.1)	7 (38.9)	0.916
Up to 11	14 (60.9)	9 (39.1)	
> 11	218 (64.3)	121 (35.7)	
Professional occupation			
With occupation	162 (66.9)	80 (33.1)	0.108
With no occupation	81 (58.7)	57 (41.3)	
Family income (wages) a			
Up to 2	194 (63.0)	114 (37.0)	0.048
> 2	61 (85.9)	10 (14.1)	
Conjugality			
No	18 (48.6)	19 (51.4)	0.041
Yes	225 (65.6)	118 (34.4)	
Use of hormone therapy			
No	229 (64.0)	129 (36.0)	0.975
Yes	14 (63.6)	8 (36.4)	
Total	243 (63.9)	137 (36.1)	

a Do not know how to answer: 1 (0.3%).

* Pearson chi-square test.

Women with a partner (OR _ad_ 2.07; 95%CI 1.03–4.17) and those with reports of depressed mood (OR _ad_ 2.03; 95%CI 1.32–3.10), sleep problems (OR _ad_ 2, 72; 95%CI 1.77–4.19), sexual problems (OR _ad_ 8.16; 95%CI 5.06–13.15) and vaginal dryness (OR _ad_ 3.44; 95%CI 2.22–5.32) showed greater chance of risk of sexual dysfunction ( Table 5 ).

**Table 5:** Multiple logistic regression for risk of sexual dysfunction associated with sociodemographic characteristics and symptoms of menopause

Variables	OR _ad_^1^	95% CI ^2^		*p* -value *
Sociodemographic characteristics				
Professional occupation	0.73	0.47	1.14	0.169
Family income	0.26	0.05	1.20	0.084
Conjugality	2.07	1.03	4.17	< 0.001
Symptoms				
Hot flashes	1.12	0.658	1.910	0.674
Cardiac malaise	1.15	0.68	1.96	0.598
Sleeping problems	2.72	1.77	4.19	0.001
Depressed mood	2.03	1.32	3.10	0.041
Irritability	1.20	0.66	2.17	0.556
Anxiety	0.86	0.47	1.58	0.623
Physical and mental exhaustion	1.38	0.77	2.47	0.274
Sexual problems	8.16	5.06	13.15	< 0.001
Bladder problems	0.97	0.49	1.92	0.931
Vaginal dryness	3.44	2.22	5.32	< 0.001
Muscle/joint problems	0.69	0.39	1.23	0.204

95%CI: 95% confidence interval; OR _ad_ : adjusted odds ratio.

* Multivariable logistic regression model.

## Discussion

The postmenopausal period is marked by physical and psychological changes typical of a woman's life. When symptomatic, this phase can have a negative impact on sexuality and, thus, be associated with sexual dysfunctions. 13 In the present study, 98.4% of the participating women had climacteric symptoms, especially urogenital symptoms. The high prevalence of climacteric symptoms was also found in a study that evaluated 1,210 women between 45 and 60 years old in São Luís, Maranhão, Brazil, present in 85.9% of the interviewees, with vaginal dryness being the most prevalent and intense symptom (68.2%). 23

The data in this study showed a high prevalence (64.0%) of sexual dysfunction. Similar findings were also observed in a survey that used the female sexual function index (FSFI) questionnaire in Natal, Rio Grande do Norte, Brazil, which found that 67% of women had some sexual dysfunction. 10 In a different way, two other studies showed a lower prevalence of dysfunction sexual. In Recife, Pernambuco, Brazil, when applying the SQ-F to 173 women aged 35 to 65 years, a prevalence of 46.2% 24 was observed. In Sergipe, Brazil, when using the FSFI instrument, a prevalence of 42.9% of sexual dysfunction was observed. 12

The FSFI is one of the most used validated psychometric scales to assess sexual function. 10 12 23 25 26 27 This instrument, validated in Portuguese, contains 19 items with multiple-choice responses to evaluate the sexual function in the 4 weeks prior to answering the questionnaire, and includes 6 domains: desire, arousal, lubrication, orgasm, satisfaction, and pain (dyspareunia). 28 Unlike the SQ-F, no domain related to the couple's relationship is present in FSFI scale. A Brazilian study with pregnant women showed that moderate-to-strong correlations were obtained between the final scores of FSFI and SQ-F, except for the comfort/pain domain. 29 However, caution is recommended when comparing results from different populations on different scales of sexual function. 30

It is known that hypoestrogenism can cause damage to the female sexual response, mainly due to the primordial role it plays on sexual desire, thus predisposing the appearance of sexual dysfunctions. 3 25 31 In a cross-sectional study, the prevalence was higher for the group of postmenopausal women (53.8%), when compared with pre-menopausal women (37.9%). 12 Other research also corroborated these results, validating the idea that the advent of menopause can have a negative impact on sexual function, especially when associated with symptoms typical of this phase of life. 26 27 32

Like other Brazilian studies, 10 24 an association was observed between all climacteric symptoms on the MRS scale and sexual function (SQ-F). Among women at risk of sexual dysfunction, there is a higher frequency of hot flashes, depressed mood, sexual problems and vaginal dryness. 10 Furthermore, there is a significant increase in sexual complaints with the intensification of climacteric symptoms. 33 There is usually an inverse correlation of climacteric symptoms assessed by MRS scale with sexual function scores, especially when analyzing urogenital symptoms. 19 In the present study, the use of hormone therapy was not associated with a lower risk of sexual dysfunction. Type, timing, and regular use of hormone therapy were not investigated, and these factors may have an influence on the results found.

The decline in ovarian function, and pituitary changes modify the pelvic tissues and the vaginal mucosa, contributing to the emergence of vaginal dryness, bleeding, and dyspareunia that can have an impact on sexual desire. 13 25 31 In the present study, sexual desire was the domain that most negatively affected the sexual function of the women interviewed. Hypoactive sexual desire is the most commonly found symptom among female sexual dysfunctions. 20 32 34 Research data with Colombian women who reported sexual activity in the 4 weeks prior to being asked showed that, also in the postmenopause, sexual desire was the most deteriorated domain. 35

A data observed in the present study was that the prevalence of sexual dysfunction in women from Teresina, detected by the application of the SQ-F (64.0%), was similar to the report of sexual problems on the MRS scale (64.7%), emphasizing that most of the women surveyed had already identified some dissatisfaction in their sex life. It is worth mentioning that the literature suggests that a sexual problem should only be considered as a dysfunction when it causes suffering in the woman's life. 34 However, some studies bring controversial results when relating the percentages of sexual dissatisfaction on the MRS scale and sexual dysfunction assessed by the SQ-F and FSFI, and sexual dissatisfaction reported by women may be lower than the percentage of sexual dysfunction detected by the FSFI and SQ-F. 24 36

The presence of conjugality was a factor that significantly increased the chance of sexual dysfunction in women from Teresina. The data available in the literature are not unanimous to support this finding. Sexual problems are very broad concepts which can be caused by biopsychosocial changes. Professional occupation, family income, and conjugality are factors that can affect the sexual response at levels of individual, interpersonal, and social influence. 37 Although many factors related to conjugality can have an impact on female sexual function, such as sexual compatibility and relationship satisfaction, this study did not make an association between conjugality and specific sexual dysfunction. 37

Women with sexual partners and a spouse were less likely to have sexual dysfunction in a study with middle-aged Brazilian women with a minimum formal education of 11 years. 9 Conversely, a cross-sectional study with 412 women in the age group of 40 to 59 years who attended health centers in Santiago, Chile, showed that married women were more likely to experience sexual dysfunction when compared with single women. 36 The study performed in the state of Pernambuco, Brazil, involving 173 women, found no association between conjugality and sexual dysfunction. 24

Some limitations of the research deserve to be pointed out. First, the women participating in the present study were selected from the users who sought health services and who attended the randomly chosen BHU. Thus, it is not possible to state that the sample may represent the general female population of Teresina, Piauí, Brazil, not covering the entire social and/or cultural variety of the general population of the city. Second, illiterate women with low schooling were included in the present study, since this is a central sociodemographic characteristic of users of the local BHU. For some participants, the questionnaire was read and filled out with the help of a researcher, and this may have generated bias. Third, sexual dysfunctions were not investigated in the participants' partnerships. Male sexual problems, for example, can influence women's sexual lives and interfere with findings of the prevalence of sexual dysfunctions. Finally, several factors interfere with the female sexual response. There are specific climacteric symptoms in the peri and postmenopausal phases. The MRS does not discriminate the somato-vegetative and psychological symptoms at specific climacteric phases. Although this study found an association between the presence of climacteric symptoms and worsening sexual function, we did not associate the symptoms of each climacteric phase with sexual function.

## Conclusion

The data showed a high prevalence of climacteric symptoms and risk of sexual dysfunction among the interviewees, with emphasis on sexual desire and interest. In addition, there was an association between all the symptoms evaluated by the MRS scale with the presence of sexual dysfunction, signaling the importance of the routine investigation of sexual activity in women with symptomatic climacteric. Investigating, informing, and properly treating these women can make the exercise of their sexualities more complete, favoring more pleasure, personal satisfaction, and quality of life.
